# Viscosity is an important factor of resistance to alcohol-based disinfectants by pathogens present in mucus

**DOI:** 10.1038/s41598-017-13732-2

**Published:** 2017-10-13

**Authors:** Ryohei Hirose, Takaaki Nakaya, Yuji Naito, Tomo Daidoji, Yohei Watanabe, Hiroaki Yasuda, Hideyuki Konishi, Yoshito Itoh

**Affiliations:** 1 Department of Molecular Gastroenterology and Hepatology, Graduate School of Medical Science, Kyoto Prefectural University of Medicine, Kyoto, Japan; 2 Department of Infectious Diseases, Graduate School of Medical Science, Kyoto Prefectural University of Medicine, Kyoto, Japan

## Abstract

Alcohol-based disinfectants play an important role in the prevention of healthcare-acquired infection (HAI). We investigated whether pathogens present in mucus acquire resistance to alcohol-based disinfectants, and elucidated the underlying mechanism. Both the resistance of influenza A virus and *Escherichia coli* to alcohol-based disinfectants or ultraviolet irradiation and the diffusion rate of ethanol were determined in artificial mucus or sputum samples obtained from 27 individuals with acute upper respiratory infection. Pathogens in mucus (artificial mucus or sputum samples) were not completely inactivated by alcohol-based disinfectants (survival rate >10%), suggesting that the alcohol-based disinfectants were ineffective. Pathogen survival and mucus viscosity were strongly correlated (correlation coefficient >0.7, P < 0.001). Additionally, the ethanol diffusion rate decreased with increasing mucus viscosity, which contributed to ethanol resistance. Pronase treatment of sputum samples significantly decreased sputum viscosity and increased the disinfectant effect (P < 0.001 for all). In contrast, complete inactivation was achieved by ultraviolet irradiation independently of mucus viscosity. Thus, mucus viscosity contributes to resistance of pathogens to alcohol-based disinfectants by decreasing the alcohol diffusion rate. These findings can provide a basis for developing new strategies, including improved disinfectants, for overcoming HAI.

## Introduction

Healthcare-acquired infections (HAIs) such as nosocomial influenza outbreak are major problems in medicine, which have yet to be resolved^1–4^. The simplest and most effective means of preventing HAI is maintaining good hand hygiene^5^.

The use of alcohol-based hand rubs (ABHRs) has spread around the world since the Guideline for Hand Hygiene in Health-Care Settings was released by the Center for Disease Control and Prevention in 2002^6^. Based on this and the 2009 World Health Organization Guideline on Hand Hygiene in Health Care^7^, most clinical settings have adopted the practice of keeping hands, which are not visibly contaminated, clean using ABHRs. However, HAI in medical facilities has not been completely prevented, which is partly due to lack of compliance among medical staff; indeed, a reduction in HAI incidence has been linked to greater compliance^5,8–11^. However, some studies suggest that there may be room for improvement in standard hand hygiene practices^12,13^.

Methods for evaluating the efficacy of disinfectants by adding a load material (e.g., foetal bovine serum or bovine serum albumin) have been established by the American Society for Testing and Materials (ASTM) and European Committee for Standardization (CEN)^14–17^. However, not only contaminating proteins but also other factors of body fluid could decrease the efficacy of alcohol-based disinfectants. Revealing the factors could enable the development of hand hygiene that is more suited to clinical needs, and could contribute to prevention of HAI.

Mucus (viscous body fluids such as sputum or nasal discharge) contain large amounts of mucin, which has a central protein core with multiple polysaccharide chains. Epithelial mucins are high-molecular weight glycoproteins that provide viscosity and gel-forming ability to mucus^18^. The thick epithelial mucus layer has a barrier function that is attributed to its high viscosity and protects the mucosal epithelium from gastric acid, digestive juice, and pathogens^19,20^. We investigated the mechanism by which the virus or viral RNA is present in faeces, and suggested that mucus (such as sputum or nasal discharge) protects virus or viral RNA from the effects of acid and digestive juices^21^. Based on these findings, we made the following hypothesis. We hypothesized that mucus can protect pathogens against alcohol-based disinfectants, as mucus plays an important role in protection from gastric acid and digestive juice in the human gastrointestinal tract.

To prove the hypothesis, this study investigated whether pathogens present in mucus such as sputum acquire resistance to alcohol-based disinfectants. We also examined the relationship between mucus viscosity and alcohol resistance in these pathogens, and elucidated the underlying mechanism.

## Results

### Protection of pathogens from disinfectants is related to viscosity of artificial mucus

We evaluated the resistance of pathogens [PR8 influenza A virus (IAV) and *Escherichia coli* (*E*. *coli*)] in artificial mucus preparations to alcohol-based disinfectants. Artificial mucus was prepared by dissolving carboxymethyl cellulose (CMC), guar gum (GG), xanthan gum (XG), or gelatin in either saline or phosphate-buffered saline (PBS). 80 w/w% ethanol (EA), 70 w/w% 2-propanol (IPA), or 60 w/w% 1-propanol (n-P) was used for the inactivation test, because these alcohol-based disinfectants are used commonly in clinical practice.

**Figure 1:**
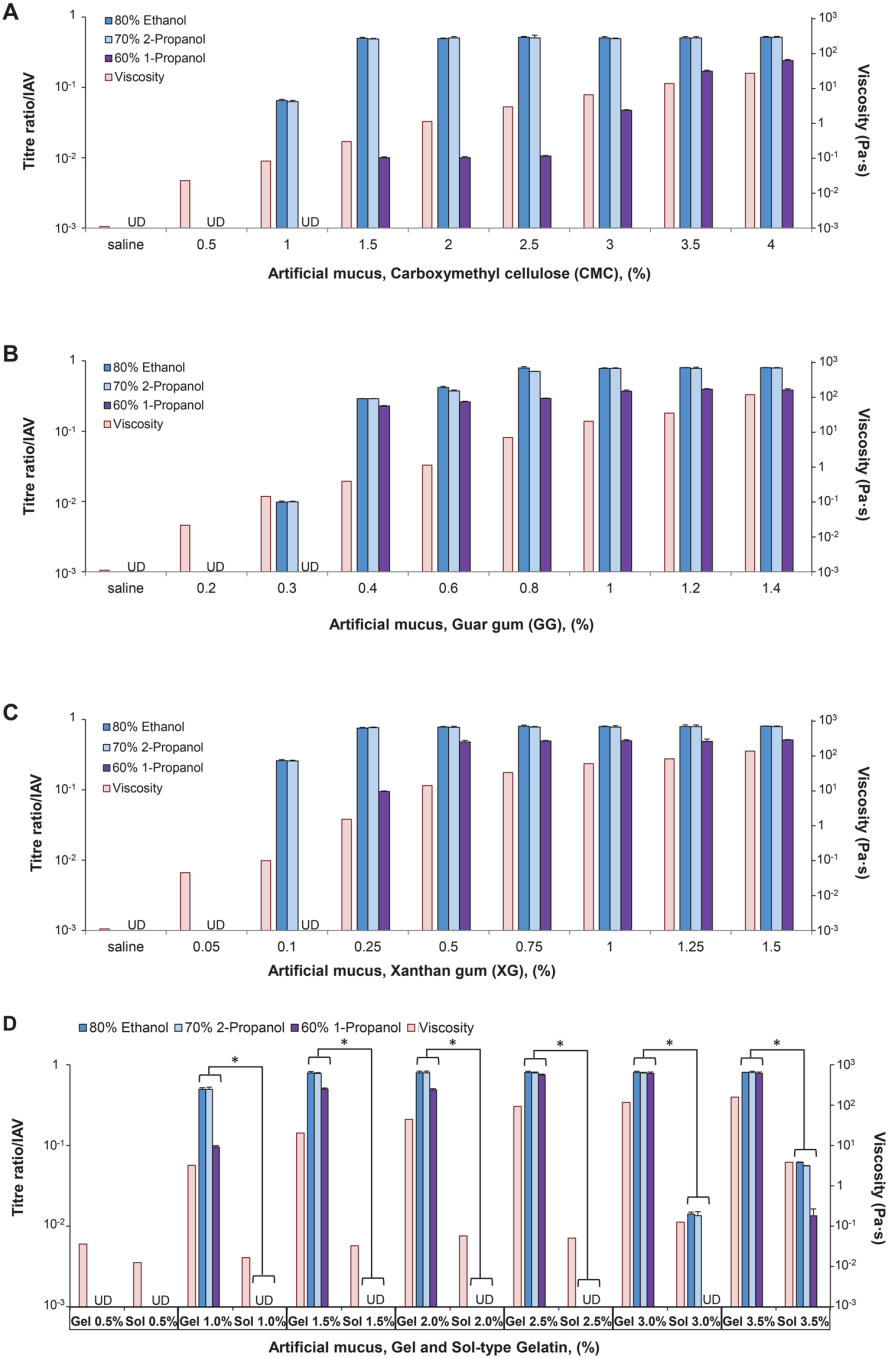
Artificial mucus protects influenza A virus (IAV) against alcoholic disinfectants. IAV [PR8, A/Puerto Rico/8/1934 (H1N1)] was mixed with artificial mucus and incubated for 30 s with alcohol-based disinfectants (80% ethanol, 70% 2-propanol, or 60% 1-propanol) before titre ratio was measured. A rheological analysis was also carried out to evaluate viscosity of artificial mucus. Viscosity of artificial mucus and titre ratios when IAV was mixed with carboxymethyl cellulose-based (**A**), guar gum-based (**B**), xanthan gum-based (**C**), and gelatin-based (**D**) artificial mucus followed by incubation with alcohol-based disinfectants. Titre ratio was defined as the ratio of the titre measured after incubation with disinfectant to the titre measured after incubation with PBS alone. The titre ratio reflected the proportion of virus (IAV) that was not inactivated by the disinfectant. Data are expressed as mean ± S.D. of at least three independent experiments. UD, undetectable. *P < 0.001.

**Figure 2:**
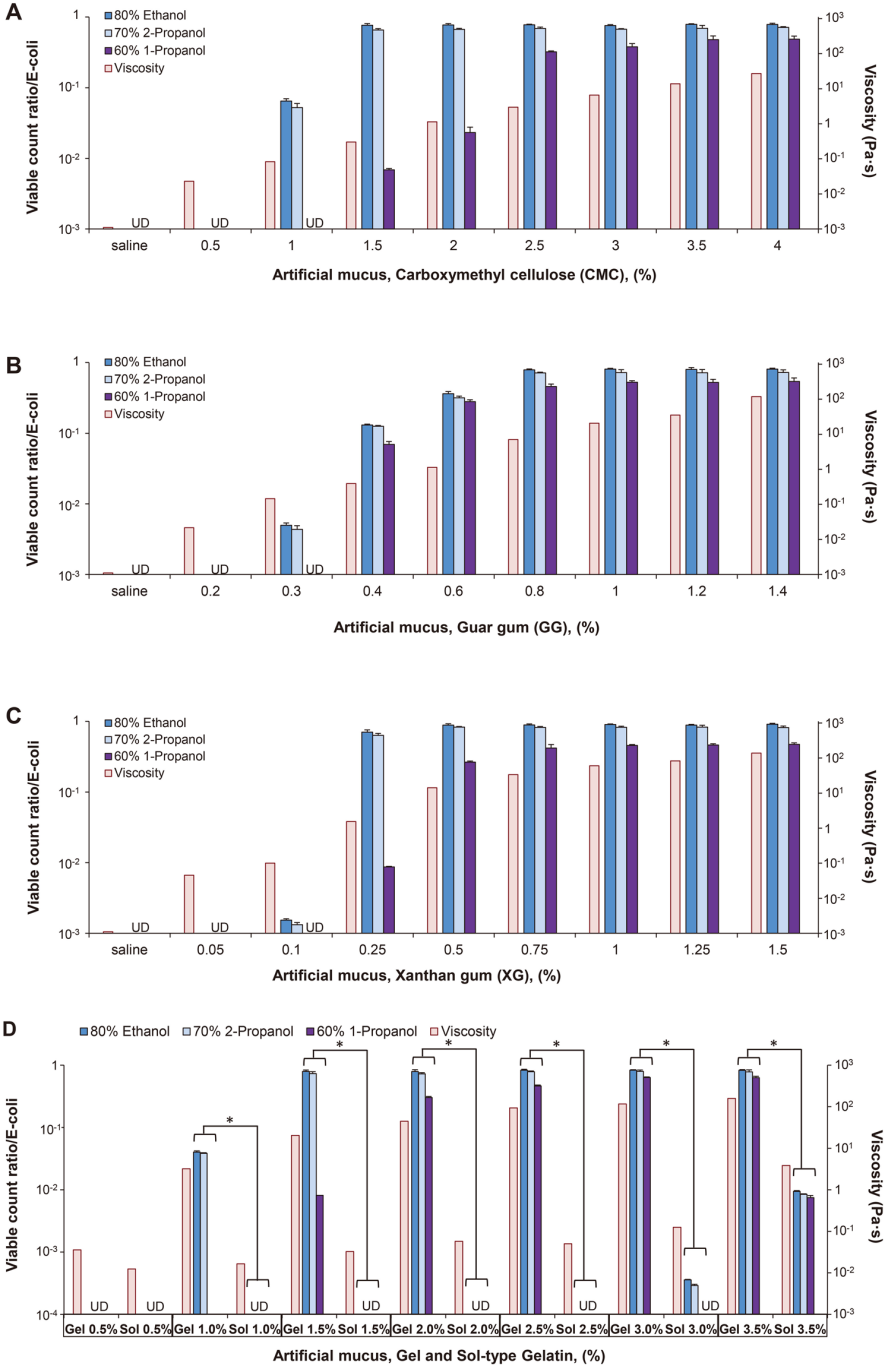
Artificial mucus protects bacteria (*E. coli*) against alcoholic disinfectants. *E*. *coli* (*E*. *coli* K12 NCTC 10538) was mixed with artificial mucus and incubated for 30 s with alcohol-based disinfectants before viable count ratio was measured. Viscosity of artificial mucus and viable count ratios when *E*. *coli* was mixed with carboxymethyl cellulose-based (**A**), guar gum-based (**B**), xanthan gum-based (**C**), and gelatin-based (**D**) artificial mucus followed by incubation with alcohol- based disinfectants. Viable count ratio was defined as the ratio of the viable count measured after incubation with disinfectant to the viable count measured after incubation with PBS alone. The viable count ratio reflected the proportion of bacteria (*E*. coli) that was not inactivated by the disinfectant. Data are expressed as mean ± S.D. of at least three independent experiments. UD, undetectable. *P < 0.001.

IAV and *E*. *coli* were completely inactivated in saline or low concentrations of CMC-, GG-, and XG-based artificial mucus. However, active (or infectious) virus and viable bacteria were detected at 1% CMC (EA, IPA) and 1.5% CMC (n-P) (Figs 1A and 2A), at 0.3% GG (EA, IPA) and 0.4% GG (n-P) (Figs 1B and 2B), and 0.1% XG (EA, IPA) and 0.25% XG (n-P) (Figs 1C and 2C), with titre/viable count ratios increasing as a function of concentration. In all types of artificial mucus, viscosity increased with mucus concentration; thus, titre/viable count ratio increased along with viscosity.

To clarify the relationship between pathogen protection from alcohol-based disinfectants and artificial mucus viscosity, pathogen resistance to disinfectants was compared between the gel and sol forms of gelatin (which differ in terms of viscosity at a given temperature) by the inactivation test. The viscosity was higher for the gel as compared to the sol form of gelatin at each concentration, and titre/viable count ratios were also significantly higher for the gel than for the sol form (≥1%) (P < 0.001 for all) (Figs 1D and 2D).

### Mucus viscosity is positively correlated with titre/viable count ratio

The above results suggest that the viscosity of artificial mucus contributes to the protection of pathogens against alcohol-based disinfectants. We created scatter plot of the viscosity of all types of artificial mucus (CMC, GG, XG and gelatin) vs. titre/viable count ratio, and used Pearson’s correlation coefficient to assess the correlation between artificial mucus viscosity and titre/viable count ratio.

**Figure 3:**
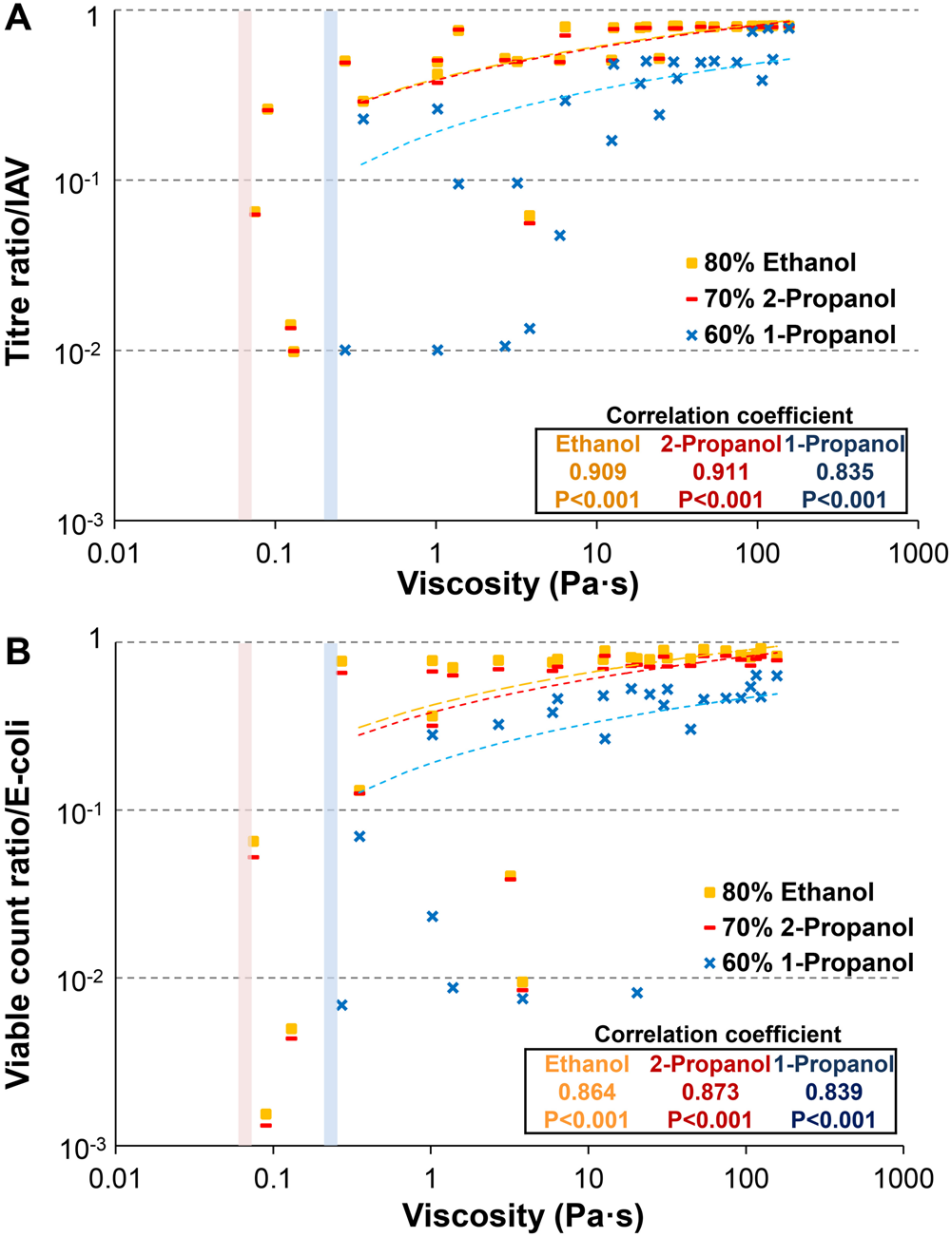
Viscosity and titre/viable count ratio are positively correlated. Scatter plot of viscosity of all types of artificial mucus vs. titre/viable count ratio; correlations were determined with Pearson’s correlation coefficient. (**A**) Pearson’s correlation coefficient between a viscosity and the titre ratio of IAV. (**B**) Pearson’s correlation coefficient between viscosity and viable count ratio of *E*. *coli*. The threshold of viscosity was approximately 0.075–0.057, below which 80% ethanol and 70% 2-propanol completely inactivated the pathogens (red line). The threshold for 60% 1-propanol was 0.27–0.13 (blue line). Below these thresholds, alcohol-based disinfectants completely inactivated pathogens.

The viscosity and titre ratio of IAV were positively correlated. The correlation coefficient was 0.909 for EA, 0.911 for IPA, and 0.835 for n-P (P < 0.001, for all) (Fig. 3A). The viscosity and viable count ratio of *E*. *coli* were also positively correlated. The correlation coefficient was 0.864 for EA, 0.873 for IPA, and 0.839 for n-P (P < 0.001 for all) (Fig. 3B).

### UV irradiation inactivates viruses and bacteria independent of viscosity

The resistance of virus to UV radiation was investigated by irradiating a mixture of IAV and artificial mucus (or saline). In saline, the titre ratio was reduced by approximately 0.01 and 0.001 at UV doses of 2.25 and 4.5 mJ/cm^2^, respectively. IAV was completely inactivated at 6.75 mJ/cm^2^. In artificial mucus, titre ratios were the same at UV doses of 2.25, 4.5, and 6.75 mJ/cm^2^, and there was no significant difference between artificial mucus and saline treatments (Supplementary Fig. S1A). The resistance of bacteria (*E*. *coli*) to UV radiation was examined in a similar manner, and there was no difference between artificial mucus and saline treatments (Supplementary Fig. S1B).

### Sputum protects pathogens against alcohol-based disinfectants

The inactivation test was also carried out using mucus from sputum samples to determine whether sputum and artificial mucus provide equal protection to pathogens against alcohol-based disinfectants.

**Figure 4:**
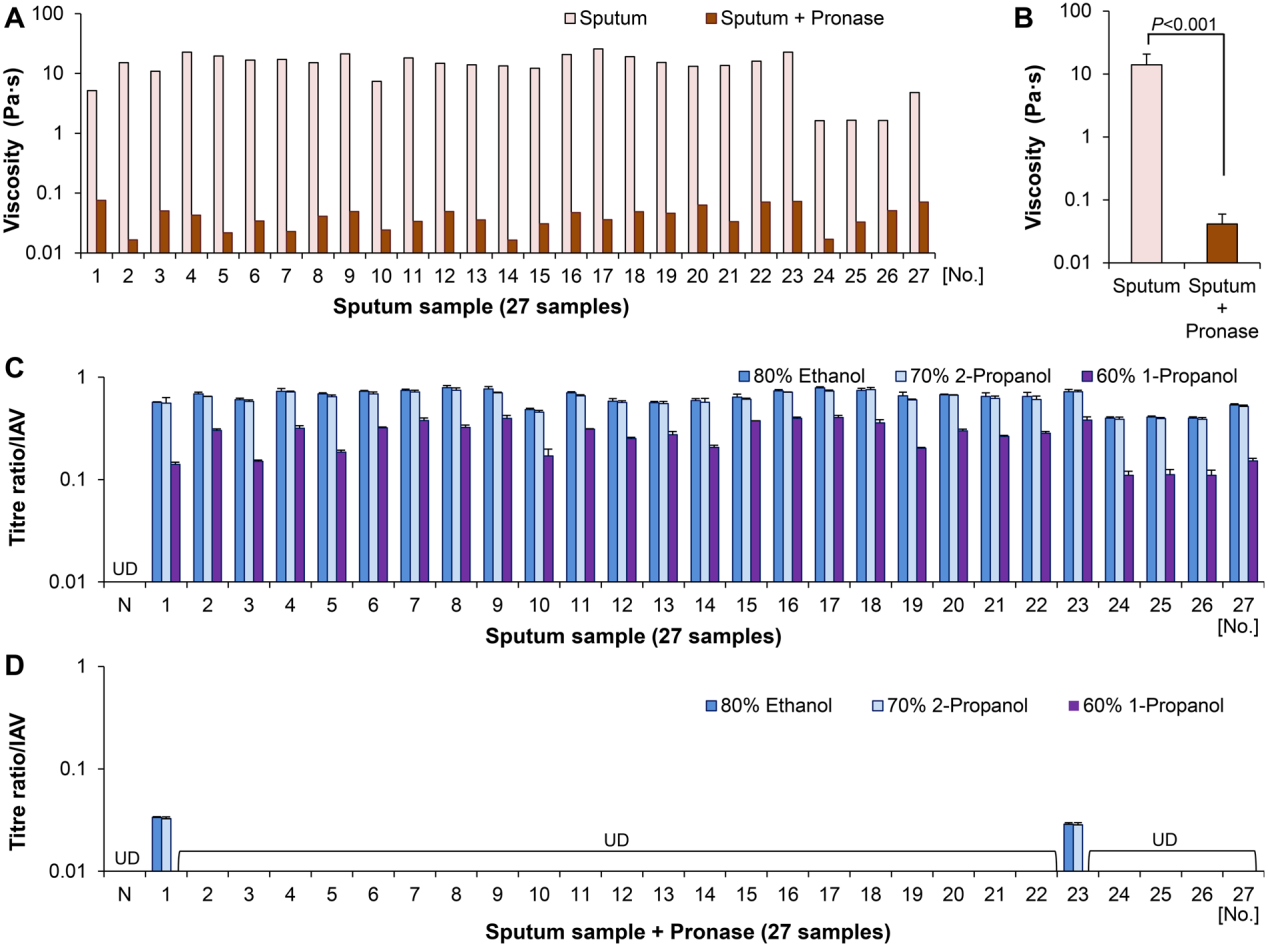
Viscosity analysis and inactivation test using sputum samples with or without pronase treatment. The inactivation test and viscosity analysis were carried out using sputum samples with or without pronase treatment and artificial mucus to determine whether pathogens are protected from alcohol-based disinfectants. (**A**) Viscosity of 27 sputum samples (with or without pronase treatment). (**B**) Comparison of sputum sample viscosity with and without pronase treatment. (**C**) Inactivation test using the 27 sputum samples to evaluate resistance to alcohol-based disinfectants. (**D**) Inactivation test using 27 sputum samples treated with pronase. N, negative control (alcohol-based disinfectants added to virus in saline); UD, undetectable. Data are expressed as the mean ± SD based on at least three independent experiments.

**Figure 5:**
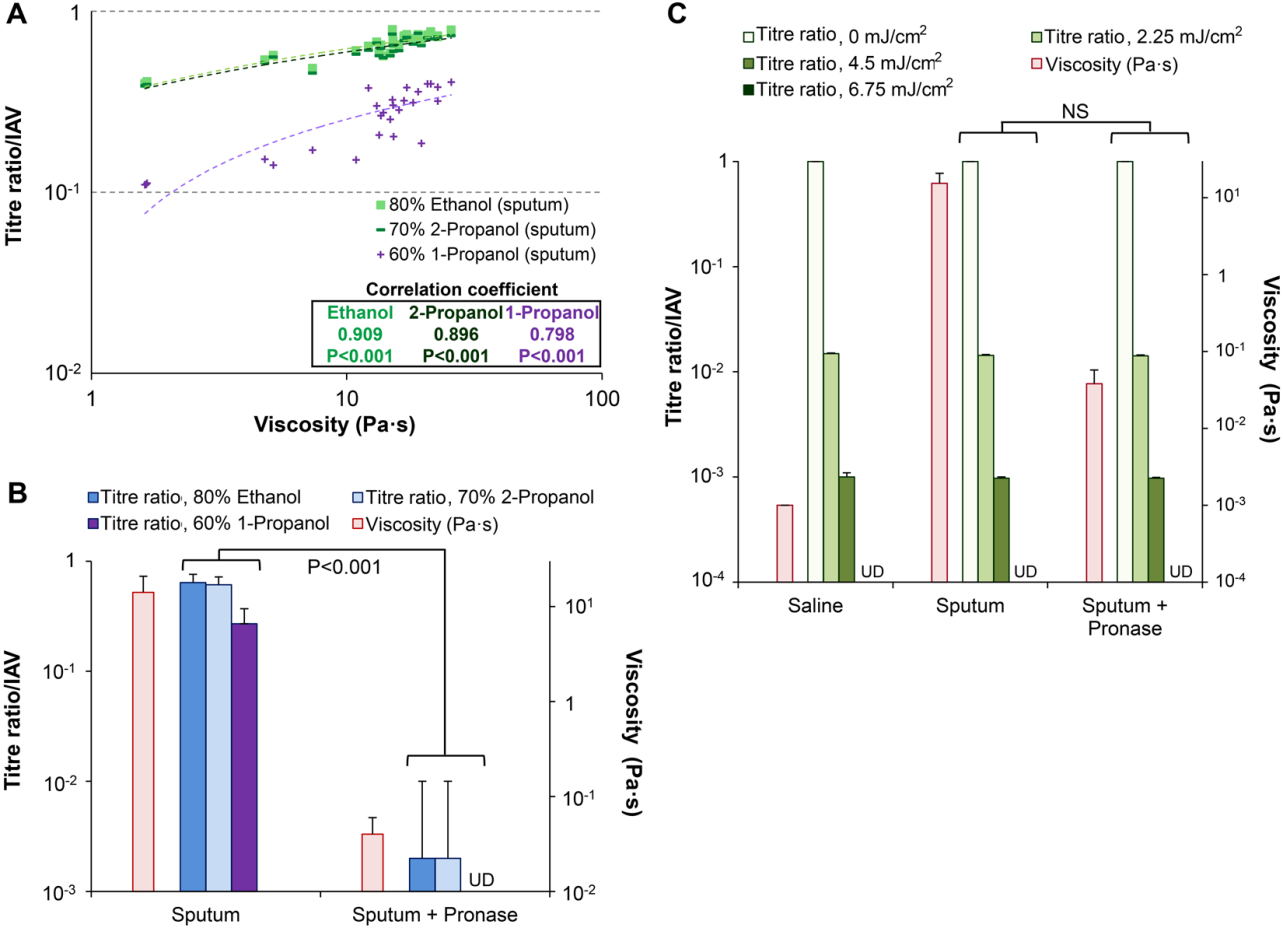
Correlation analysis and comparison with pronase-treated and untreated sputum samples. (**A**) Scatter plot of viscosity vs. titre ratio of 27 sputum samples. Pearson’s correlation coefficient was determined to assess the correlation between the two variables. (**B**) Titre ratios were compared between pronase-treated and untreated groups based on inactivation test results. (**C**) Resistance to UV radiation (0, 2.25, 4.5, or 6.75 mJ/cm^2^) of IAV in sputum samples with or without pronase treatment. UD, undetectable; NS, not significant. Data are expressed as the mean ± SD based on at least three independent experiments.

The viscosity analysis of 27 sputum samples revealed mean and median viscosities of 14.1 ± 6.7 Pa·s (range: 1.62–25.7 Pa·s) and 15.1 Pa·s (interquartile range: 11.5–18.6 Pa·s), respectively (Fig. 4A). The results of the inactivation test showed that infectious virus (IAV) was present in all 27 sputum samples after incubation with alcohol-based disinfectants (Fig. 4C and Supplementary Table S1). The scatter plot of viscosity vs. titre ratio yielded Pearson’s correlation coefficients of 0.909 for EA, 0.896 for IPA, and 0.798 for n-P (P < 0.001 in each case) (Fig. 5A). The scatter plots for tests performed on artificial mucus and sputum samples were merged (Figs 3A and 5A) to compare the protective effects of the two types of mucus. The results showed that sputum samples protected pathogens from alcohol-based disinfectants as effectively as artificial mucus (Supplementary Fig. S2), and that the titre ratio was determined by viscosity independent of mucus type.

Next, pathogen resistance to alcohol-based disinfectants was compared between sputum samples without or with pronase treatment to decrease mucus viscosity, after first confirming that IAV was not inactivated by pronase treatment alone. The mean viscosity was 0.04 ± 0.02 Pa·s for the 27 pronase-treated sputum samples (Fig. 4A), whereas the results of the inactivation test showed that almost all viruses were inactivated (Fig. 4D and Supplementary Table S2). The mean viscosity was lower for samples with than for those without pronase treatment (0.04 ± 0.02 vs. 14.1 ± 6.7, respectively; P < 0.001) (Fig. 4B). The mean titre ratios of the pronase-treated sputum group were 0.002 ± 0.008 (EA), 0.002 ± 0.008 (IPA), and 0.00 ± 0.00 (n-P); these values were significantly lower than those of untreated samples (0.64 ± 0.12, 0.61 ± 0.11, and 0.27 ± 0.10, respectively; P < 0.001 in each case) (Fig. 5B and Supplementary Table S3). These results indicate that protection of pathogens against alcohol-based disinfectants is related to sputum viscosity.

We also evaluated the resistance of IAV in sputum samples to UV radiation. No difference was found in titre ratios between sputum with and without pronase treatment at UV doses of 2.25, 4.5, and 6.75 mJ/cm^2^ (Fig. 5C and Supplementary Table S4), suggesting that sputum viscosity did not affect the inactivating effect of UV.

### Inactivation of virus by disinfectants is time-dependent

**Figure 6:**
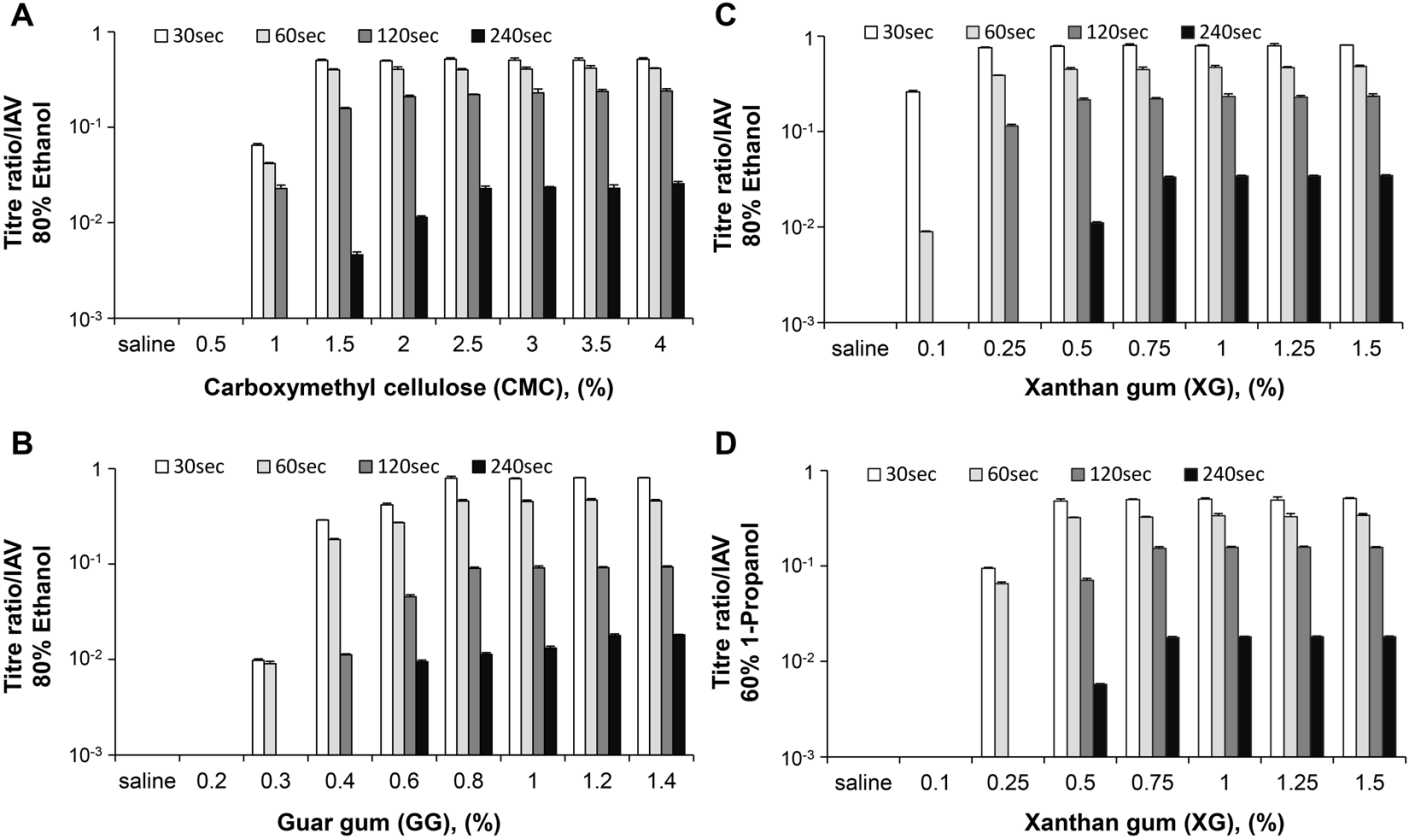
Effects of alcohol-based disinfectants over time on viral titre ratios. IAV was mixed with artificial mucus and incubated for 30, 60, 120, and 240 s with alcohol-based disinfectants (80% ethanol or 60% 1-propanol) before viral titre ratio was measured. (**A**–**C**) Titre ratios of IAV mixed with carboxymethyl cellulose-based (**A**), guar gum-based (**B**), and xanthan gum-based (**C**) artificial mucus followed by incubation with 80% ethanol. (**D**) Titre ratios of IAV mixed with xanthan gum-based artificial mucus and incubated with 60% 1-propanol. Data are expressed as the mean ± SD based on at least three independent experiments.

Time-dependent effects of alcohol-based disinfectants (EA or n-P) on IAV in artificial mucus preparations were examined. When CMC, GG, or XG virus preparations were treated with EA for 30, 60, 120, or 240 s, the titre ratio decreased over time (Fig. 6A–C). A similar trend was observed for XG-based artificial mucus virus preparations treated with n-P (Fig. 6D). These results suggest that the disinfectant effects of alcohol were not completely abolished but were delayed by artificial mucus.

### Diffusion rate of ethanol decreases with increased mucus viscosity

**Figure 7:**
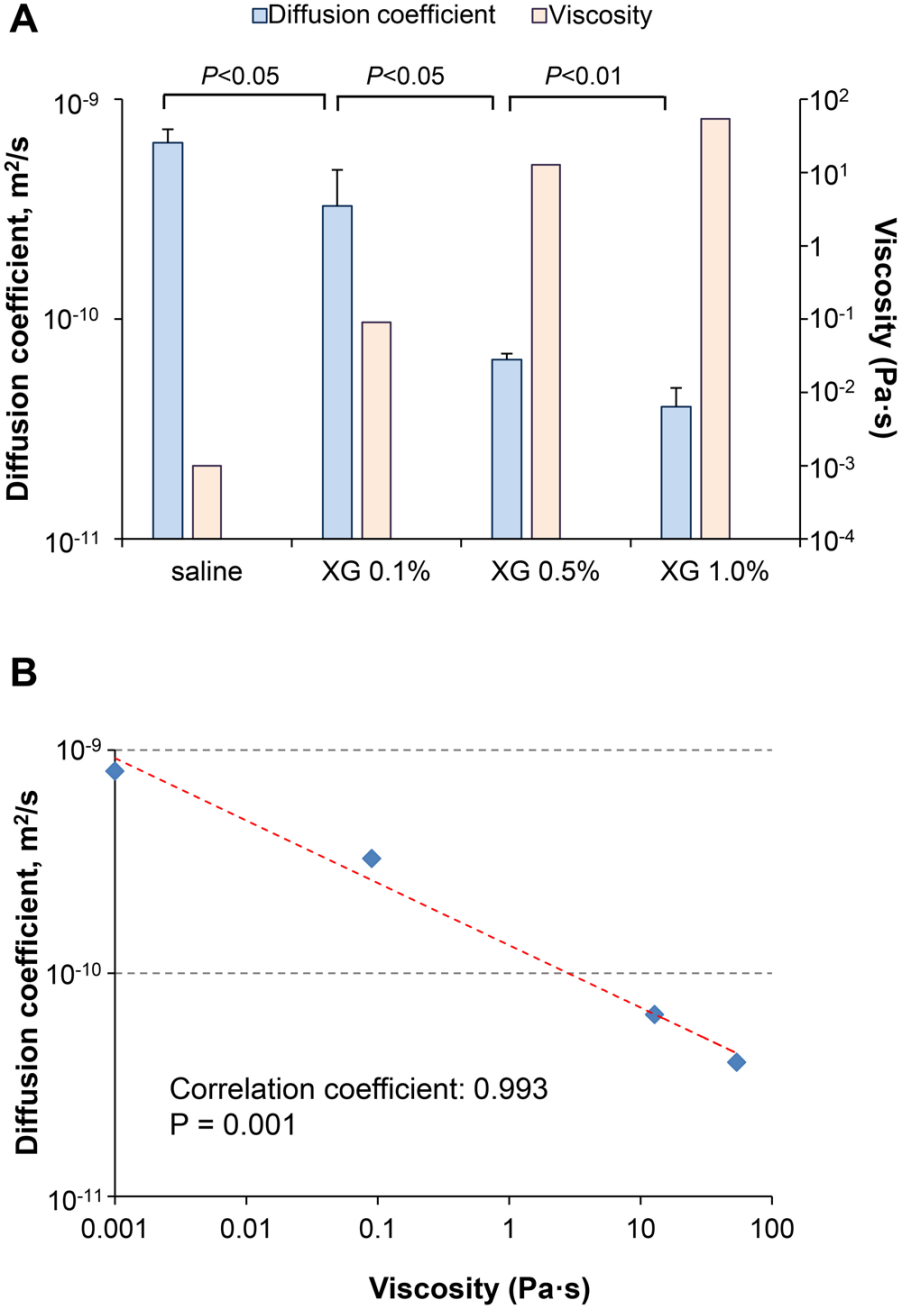
Evaluation of the diffusion rate of ethanol in saline and artificial mucus. (**A**) The rate (diffusion coefficient) at which ethanol molecules diffuse into xanthan gum-based artificial mucus or saline was determined (diffusion coefficient, light blue bar; viscosity, light red bar). (**B**) Scatter plot of the viscosity of artificial mucus vs. diffusion coefficient; correlations were determined using the Pearson’s correlation coefficient. XG, xanthan gum. Three independent experiments were performed and the results are expressed as the mean ± standard deviation.

Alcohol molecules must permeate a target via diffusion in order to exert their effects. The diffusion rate (interdiffusion coefficient) at which ethanol molecules diffuse into mucus was determined. The ethanol diffusion coefficient in the artificial mucus condition (0.1% XG) was lower than that in the saline condition (6.35 × 10^−10^ ± 9.44 × 10^−10^ m^2^/s vs. 3.27 × 10^−10^ ± 1.51 × 10^−10^ m^2^/s, P = 0.0398). Additionally, the diffusion coefficient significantly decreased with increasing viscosity of the artificial mucus (Fig. 7A). The viscosity and the diffusion coefficient were negatively correlated, with a correlation coefficient of 0.993 (P = 0.001) (Fig. 7B).

## Discussion

The major findings of the present study are as follows. (i) Pathogens present in artificial mucus were not completely inactivated by alcohol-based disinfectant; titre/viable count ratios increased with viscosity of artificial mucus. (ii) The inactivation test revealed a positive correlation between titre/viable count ratio and mucus viscosity. (iii) The ethanol diffusion coefficient decreased with increasing mucus viscosity. (iv) UV irradiation completely inactivated pathogens, which was independent on viscosity. (v) Viruses in sputum samples were protected from alcohol-based disinfectant, and the correlation between sputum viscosity and titre ratio was similar to that determined using artificial mucus. (vi) Pronase treatment decreased sputum viscosity and increased the disinfectant effect.

To simulate viscous body fluids such as sputum, we created artificial mucus, using four types of solute (CMC, GG, XG, and gelatin) differing in terms of viscoelastic behaviour. The artificial mucus with different viscoelastic behaviours allowed us to exclude confounding factors (e.g., solute properties) and accurately analyse the correlation between viscosity and titre/viable count ratio. The protection of pathogens by artificial mucus was independent of solute properties, but was strongly correlated with viscosity. This was examined in greater detail using gel and sol forms of gelatin. Between two types of artificial mucus differing in terms of viscosity but composed of the same solutes in the same amounts (gelatin gel and sol), the one with higher viscosity (i.e., the gel form) provided greater protection to pathogens against alcohol-based disinfectants.

We analysed the relationship between reaction time with alcohol-based disinfectants and titre ratio. In artificial mucus, longer reaction times resulted in lower titre ratios, suggesting that mucus did not render alcohol ineffective; instead, alcohol retained its ability to inactivate pathogens, but the effects were slowed.

Alcohol molecules must permeate a target (infected body fluid) via diffusion and convection (hand-rubbing action) in order to exert their effects. In the dilute solution condition, according to the Stokes-Einstein equation (D = kT/6πηR), diffusion rate (diffusion coefficient, D) decreases with increases in target viscosity (η)^22–26^. In our results, the ethanol diffusion coefficient in mucus (low concentration artificial mucus or sputum samples) was lower than that in saline, and the diffusion coefficient decreased with increasing mucus viscosity.

When either diffusion or convection decreases, alcohol requires more time to exert its effects throughout the target. Under the same convective (hand-rubbing action) conditions, a 10-fold decrease in ethanol diffusion coefficient leads to a 10-fold increase in the time required for ethanol to reach an effective concentration (complete inactivation of pathogens). When the viscosity of an environment containing pathogens increases, the diffusion coefficient of alcohol decreases, thereby extending the time needed for complete inactivation of pathogens. When a standard alcohol reaction time (30 s) is used^6,7^, some pathogens survive, because more time is needed for their complete inactivation. Thus, viscosity is an important factor of alcohol resistance by pathogens present in mucus.

We investigated whether IAV in sputum samples are protected from the effects of alcohol-based disinfectants; this model reflects the sputum of individuals infected with influenza virus. Although previous studies have reported that IAV is vulnerable to alcoholic disinfectants^27^, the present study showed that IAV present in sputum was not completely inactivated by alcohol-based disinfectants. The conditions of the inactivation test (i.e., amount of pathogen, alcohol ratio, and reaction time) were in accordance with established standards; that is, disinfectants were deemed effective when the titre ratio was ≤10^−4^ ^14–17^. In the inactivation test, titre ratios were approximately 10^−1^, suggesting that IAV in sputum may acquire the resistance to alcohol-based disinfectants. There was a strong correlation between sputum viscosity and titre ratio; indeed, when scatter plots for tests performed on artificial mucus and sputum samples were merged, the data showed similar trends, implying that the same correlation existed for artificial mucus and sputum.

Moreover, our study showed that pronase treatment of sputum samples decreased viscosity and increased the disinfectant effect, suggesting that viscosity is an important factor of resistance to alcoholic disinfectants by pathogens present in sputum.

On the other hand, disinfectant methods such as UV that do not require diffusion throughout the entire target to be effective may be able to completely inactivate pathogens in viscous mucus. In fact, UV irradiation completely inactivated IAV present in sputum samples, and no difference was found in titre ratios between sputum with or without pronase treatment. These results indicate that the inactivating effect of UV on sputum is not affected by viscosity.

Our study suggests that pathogens present in mucus may acquire the resistance to alcohol-based disinfectants. In the current ASTM and CEN assessment standards for disinfectant effectiveness, protein loading has been shown to weaken the effectiveness of disinfectants^16,17,28,29^, but the rheology of the environment containing the pathogens (mucus viscosity) should also be considered. Current hand hygiene guidelines recommend washing with water as the first choice for removing visible contamination^6,7^. However, fine sprays of sputum or nasal fluids on hands often go unnoticed and may contain infectious substances. In this study, such sprays were mimicked in inactivation tests using small amounts (15–30 µl) of pathogen-containing mucus. Since such sprays may acquire the resistance to alcohol-based disinfectants, hand washing that washes out mucus physically remains the preferred practice for good hand hygiene. Disinfectant methods such as UV that do not require diffusion throughout the entire target to be effective are able to inactivate pathogens in viscous liquids. Such methods deserve more study in the future. Moreover, considering the presence of viscous infectious substances as part of the current ASTM and CEN assessment standards for disinfectant effectiveness can provide a basis for developing disinfectants that are more suited to clinical needs and can prevent HAI.

A limitation of this study was that the influence of contaminants in sputum samples was not evaluated, although the viscosity of the sputum samples was measured. Contaminants in sputum samples may reduce the effects of alcohol-based disinfectants. However, because the titre ratio/viable count ratio were almost equivalent under various artificial mucus conditions and sputum conditions, we suggest that the influence of contaminants was small and that viscosity was the important factor in pathogen protection. Furthermore, when alcohol-based disinfectant is added to pathogen/artificial mucus and incubated, it is difficult to normalise the conditions of admixture with alcohols, which results in low reproducibility. In other words, because of the instability of convection (mixing) conditions, the inactivation/sterilization reaction may not be stable. In this study, the inactivation/sterilization reaction was stable and reproducibility was maintained because a single experimenter conducted the experiments using a similar procedure. Although our conclusion that the titre ratio/viable count ratio increased as the viscosity increased remains valid, other groups may obtain slightly different results when repeating these experiments.

In conclusion, our study demonstrated that mucus viscosity contributes to resistance of pathogens to alcohol-based disinfectants. Our findings will lead to the development of more effective products and practices for preventing HAIs. Phase II clinical trials for evaluating *in vivo* the effectiveness of disinfectants in mucous environments are currently underway.

## Methods

### Cell, virus, and bacteria

Madin-Darby canine kidney cells (MDCK) were purchased from the Riken BioResource Center Cell Bank (Ibaragi, Japan) and cultured in minimal essential medium (MEM; Sigma, St. Louis, MO, USA) supplemented with 10% foetal bovine serum and standard antibiotics^30,31^. IAV [PR8; A/Puerto Rico/8/1934 (H1N1)] was cultured in MDCK cells, and stored as a working stock at −80 °C. Viral titre was measured by focus-forming assays in MDCK cells and expressed as the number of focus-forming units (FFU)/ml. *E*. *coli* K12 NCTC 10538 was purchased from the National Collection of Type Cultures (Salisbury, England) and cultured on lysogeny broth. Bacterial viable count was measured with a colony-forming assay in mannitol salt agar and is expressed as the number of colony-forming units (CFU)/ml.

### Preparation of artificial mucus

Artificial mucus was prepared by dissolving carboxymethyl cellulose (CMC; Wako Pure Chemical Industries, Osaka, Japan), guar gum (GG; Wako Pure Chemical Industries), xanthan gum (XG; Tokyo Chemical Industry, Tokyo, Japan), or gelatin (Nacalai Tesque, Kyoto, Japan) in either saline or phosphate-buffered saline (PBS). CMC artificial mucus was prepared at concentrations of 0.5%, 1.0%, 1.5%, 2.0%, 2.5%, 3.0%, 3.5%, and 4.0% (w/v); GG artificial mucus was prepared at concentrations of 0.2%, 0.3%, 0.4%, 0.6%, 0.8%, 1.0%, 1.2%%, and 1.4% (w/v); XG artificial mucus was prepared at concentrations of 0.05%, 0.1%, 0.25%, 0.5%, 0.75%, 1.0%, 1.25%, and 1.5% (w/v); and gelatin artificial mucus was prepared at concentrations of 0.5%, 1.0%, 1.5%, 2.0%, 2.5%, 3.0%, and 3.5% (w/v) and were of two types (sol and gel). For the former, gelatin was dissolved in PBS at 60 °C and cooled to 20 °C; for the latter, gelatin was dissolved in PBS at 60 °C and cooled to 4 °C for gelling, and then warmed to 20 °C before use.

CMC is a cellulose derivative with carboxymethyl groups bound to some of the hydroxyl groups of the glucopyranose monomers that make up the cellulose backbone. GG is a polysaccharide composed of the sugars galactose and mannose.

XG is a viscoelastic extracellular polymeric substance (EPS), with a high molecular weight, and is produced by the bacterium *Xanthomonas campestris*. It is a hetero-polysaccharide with repeated pentasaccharide units consisting of two molecular structures of glucose and mannose and one unit of glucuronic acid^32^. Gelatin is a mixture of peptides and proteins produced by partial hydrolysis of collagen extracted from tissues of animals. These solutes of artificial mucus are generally used to increase the viscosity of water-based products (food or drugs).

### Collection of mucus (sputum samples)

A prospective observational study designed to evaluate the viscosity of sputum samples from individuals with acute upper respiratory infection was carried out. The study protocol was reviewed and approved by the Institutional Review Board of the Kyoto Prefectural University of Medicine (ERB-C-634). All experiments of this research were performed in accordance with relevant guidelines and regulations. Sputum samples (1600 mg) were obtained from individuals diagnosed with acute upper respiratory infection at the Department of General Medicine of Kyoto Prefectural University of Medicine Hospital between August 2016 and September 2016. Minors (<20 years old), influenza-infected individuals (diagnosed by the rapid antigen detection test), patients with chronic respiratory illness, and patients taking expectorants were excluded. Informed consent was obtained at the time of examination, and 27 patients were ultimately included in the analysis.

After rheological analysis of viscosity, a part of each sputum sample (800 mg) was stored at 4 °C for evaluation of disinfectant effects and 200 U pronase (Kaken Pharmaceutical, Tokyo, Japan) were added to the remaining sample (800 mg) to degrade mucin and thereby reduce the viscosity. The rest of the sputum sample was incubated at 37 °C for 30 min and viscosity was measured. After rheological analysis, the pronase-treated sputum sample was stored at 4 °C for evaluation of alcohol-based disinfectant effect. Pronase, mixture of proteolytic enzymes, decomposes mucin and reduces the viscosity of mucus^33,34^.

### Rheological analysis

Viscosity was measured using an AR 2000 controlled-stress rheometer (TA Instruments, Surrey, UK)^35^. A 20-mm corn plate geometry was used at 20 °C. A solvent trap containing distilled water prevented sample dehydration during measurement. Sputum samples or artificial mucus (750 mg) was loaded onto the rheometer for 15 min to allow relaxation to the original gel structure. Initial experiments were performed in dynamic oscillatory mode, which included a strain sweep (0.01–50%) to determine the linear viscoelastic region (0.01–10% for each tested sample). A frequency sweep (0.046–100 rad/s) was performed at 2%, and the rheological parameters of elastic modulus (elasticity) (G’, Pa), loss modulus (G”, Pa), and dynamic viscosity (η’, Pa·s) were determined in the linear viscoelastic region of each sample using Rheology Advantage data analysis software (TA Instruments) for a range of oscillation frequencies (0.046, 0.1, 0.22, 0.46, 1.0, 2.2, 4.6, 10, 22, 46, and 100 rad/s). We used dynamic viscosity (0.1 rad/s) as an indicator of mucus viscosity.

### Inactivation test for evaluation of disinfectant efficacy against viruses and bacteria

80 w/w% ethanol (EA), 70 w/w% 2-propanol (IPA), or 60 w/w% 1-propanol (n-P) was used for inactivation test, because these alcohol-based disinfectants are used commonly in clinical practice.

The resistance of viruses to alcohol-based disinfectants (EA, IPA, n-P) was examined with the inactivation test, which was conducted according to established guidelines (EN14476:2013/FprA1:2015)^17,28,36–38^. Virus (IAV) was mixed with saline or artificial mucus (CMC, GG, XG, or gelatin) prior to exposure to alcohol-based disinfectants. Briefly, 135 µl of disinfectant or PBS were added to 15 µl of virus/artificial mucus (final viral titre: 1.0 × 10^6^ FFU/ml) in 24-well plates and incubated at 20 °C for 30, 60, 120, or 240 s prior to neutralization with 1350 µl of MEM; the virus was then titrated. The test was also conducted with sputum samples with or without pronase treatment.

The resistance of *E*. *coli* to alcohol-based disinfectants (EA, IPA, n-P) was assessed according to ASTM or CEN guidelines (ASTM E2315, EN 13727)^15,16,29,39^. Bacterial cells were mixed with saline or artificial mucus (CMC, GG, XG, or gelatin) prior to exposure to alcohol-based disinfectants. Briefly, 135 µl of the alcohol-based disinfectants or PBS were added to 15 µl of bacteria/artificial mucus (final viable count: 2.0 × 10^6^ CFU/ml) in 24-well plates and incubated at 20 °C for 30 s before neutralization with 1350 µl MEM.

Titre/viable count ratio was determined as the ratio of titre/viable count after incubation with disinfectant to that measured after incubation with PBS only (measurements were obtained using artificial mucus preparations of different concentrations or PBS only), and reflected the proportion of virus/bacteria that was not inactivated by the disinfectant. A titre/viable count ratio closer to 1 indicated greater resistance to disinfectants. Three independent experiments were performed and the results are expressed as the mean ± standard deviation.

### Evaluation of efficacy of ultraviolet (UV) radiation against viruses and bacteria

The resistance of virus and bacteria to UV radiation was examined^40–42^. Virus (IAV) was mixed with saline or artificial mucus (CMC, GG, XG, or gelatin) prior to UV radiation exposure. A 30-µl volume of virus/artificial mucus (final virus titre; 5.0 × 10^6^ FFU/ml) was irradiated at a dose of 0, 2.25, 4.5, or 6.75 mJ/cm^2^ before dilution with 1350 µl of MEM. The virus was then titrated. The test was also conducted with sputum samples with or without pronase treatment.

*E*. *coli* cells were mixed with saline or artificial mucus, and 30 µl of bacteria/artificial mucus (final viable count: 1.0 × 10^7^ CFU/ml) were irradiated at a dose of 0, 1.13, 2.25, or 3.38 mJ/cm^2^ before dilution with 1350 µl MEM. The viable count was then determined.

The titre/viable count ratio was defined as the ratio of titre/viable count measured after to that measured before exposure to UV. Three independent experiments were performed and the results are expressed as mean ± standard deviation.

### Evaluation of the diffusion rate of ethanol in saline and artificial mucus

The rate (diffusion coefficient) at which ethanol molecules diffuse into mucus was determined. A polyethylene container with two chambers (A and B) was used in the experiment (Supplementary Fig. S3). Changes in ethanol concentration in chambers A and B due to diffusion were measured to calculate the diffusion coefficient. Chamber A was filled with ethanol (0.435 kmol/m^3^), and chamber B was filled with artificial mucus (which was prepared by dissolving 0.1%, 0.5%, and 1.0% XG in saline), saline, or water. After 120 minutes of diffusion at 20 °C, the ethanol concentration in each chamber was measured by a digital ethanol densitometer (ATAGO, Tokyo, Japan), and the diffusion coefficient calculated^22–26^. Pearson’s correlation coefficient was used to assess the correlation between viscosity and diffusion coefficient. Three independent experiments were performed and the results are expressed as the mean ± standard deviation.

### Statistical analysis

Data were analysed using GraphPad Prism 7 software (GraphPad Inc., La Jolla, CA, USA). Continuous variables were evaluated by the Student’s t test. Pearson’s correlation coefficient was used to assess the correlation between viscosity and titre/viable count ratio. Viscosity was converted to a logarithm scale and used for correlation analysis. All reported P values are two-sided and those <0.05 were considered significant.

## Electronic supplementary material

Supplementary figures and tables
