# Supplementary material for: Viscosity is an important factor of resistance to alcohol-based disinfectants by pathogens present in mucus
Source: Sci Rep. 2017 Oct 13;7:13186. doi: 10.1038/s41598-017-13732-2 (PMC5640602; doi:10.1038/s41598-017-13732-2)
Supplement: Supplementary file 1 — Supplementary figures and tables [file 41598_2017_13732_MOESM1_ESM.pdf]

Supplementary information (Supplementary figures and tables)

Viscosity is an important factor of resistance to alcohol-based disinfectants by pathogens present in mucus

Ryohei Hirose, Takaaki Nakaya, Yuji Naito, Tomo Daidoji, Yohei Watanabe, Hiroaki Yasuda, Hideyuki Konishi, Yoshito Itoh

Table of Contents

1. Supplementary Figure S1.

2. Supplementary Figure S2.

3. Supplementary Figure S3.

4. Supplementary Table S1.

5. Supplementary Table S2.

6. Supplementary Table S3.

7. Supplementary Table S4.

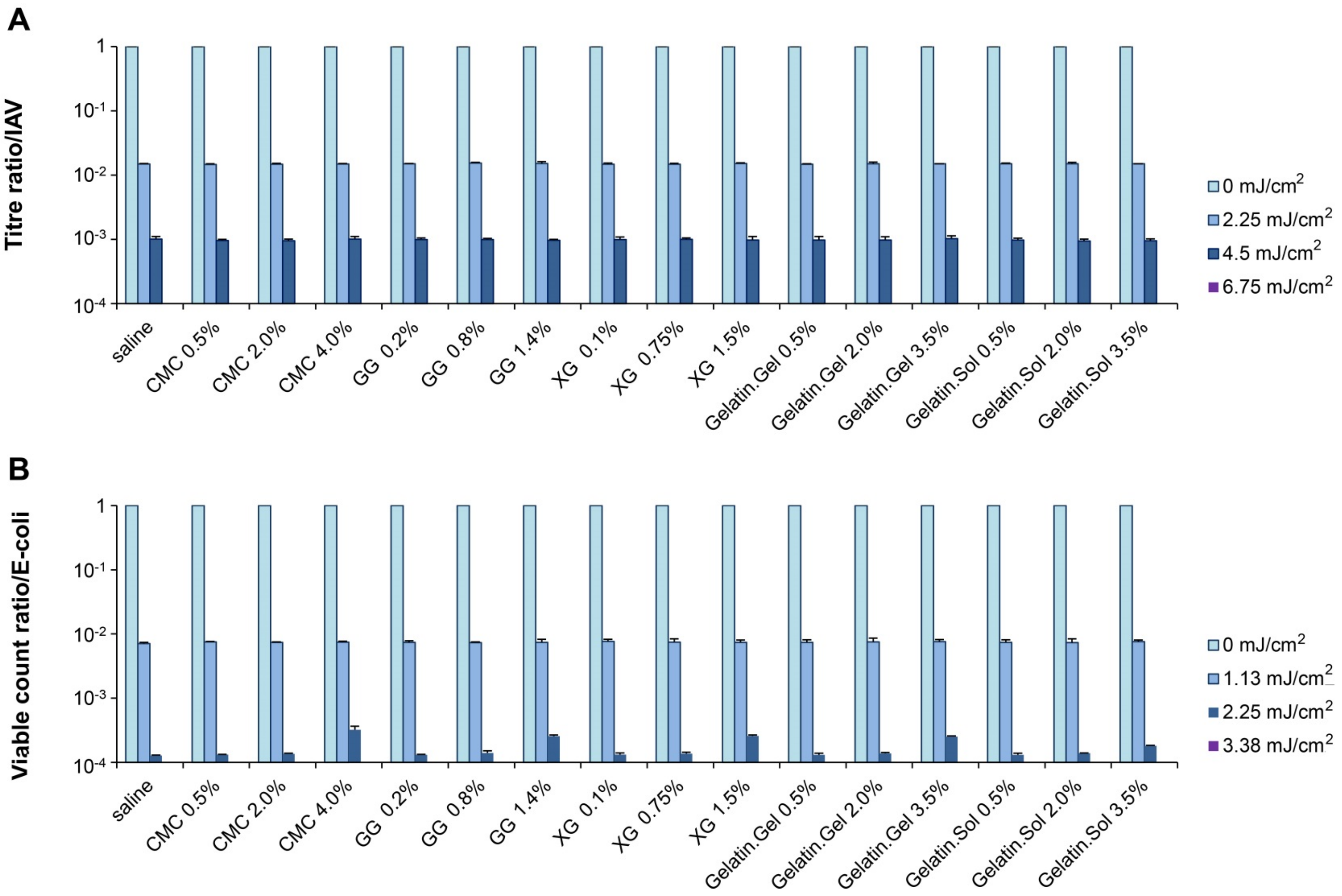

Supplementary Figure S1. Effect of UV radiation on virus and bacteria. (A) IAV was mixed with saline or artificial mucus (CMC, carboxymethyl cellulose; GG, guar gum; XG, xanthan gum; or gelatin gel/sol) and then exposed to UV radiation (0, 2.25, 4.5, or 6.75 mJ/cm^2) before titre ratio was measured. (B) *E. coli* cells were mixed with saline or artificial mucus and then exposed to UV radiation (0, 1.13, 2.25, or 3.38 mJ/cm^2) before viable count ratio was determined.

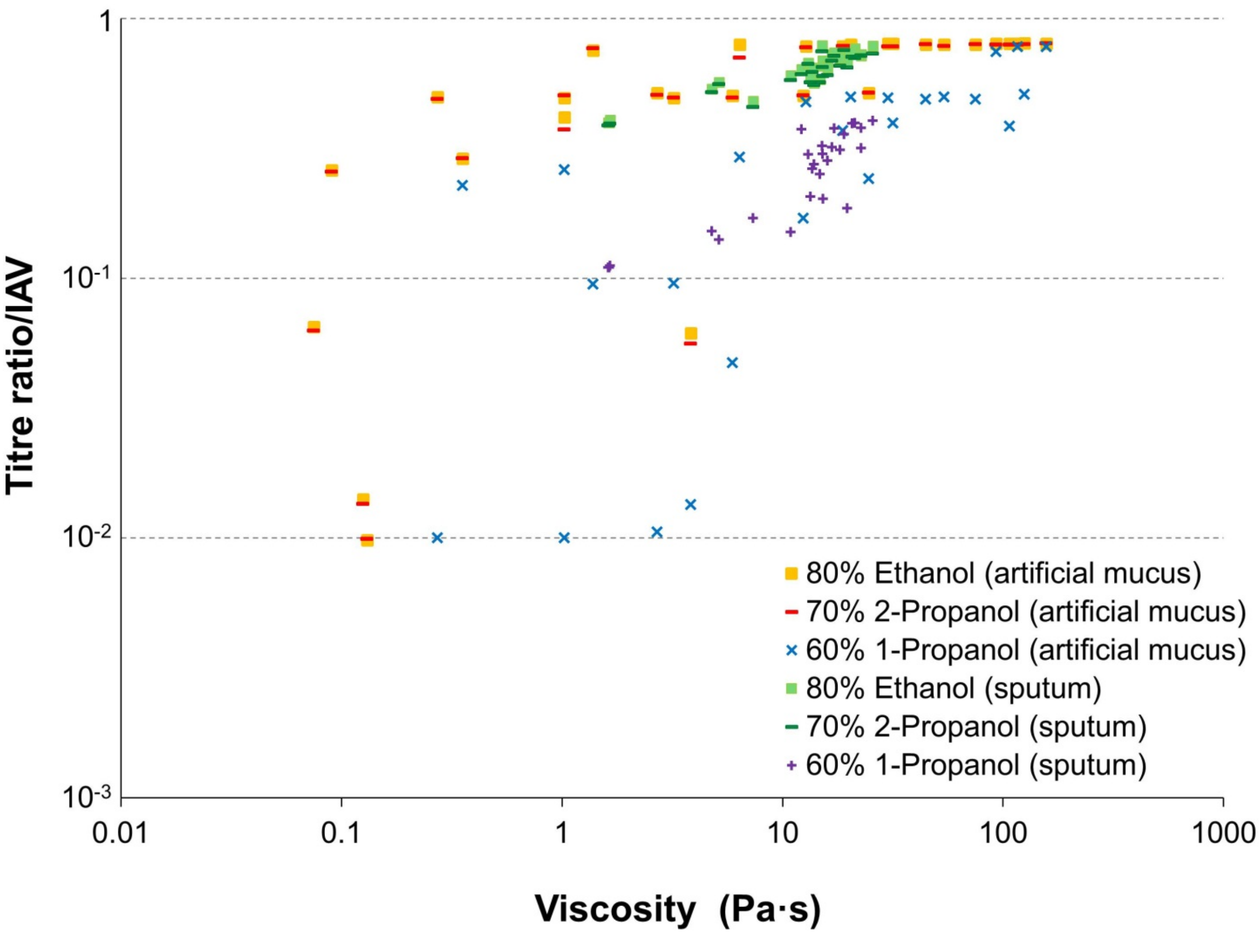

Supplementary Figure S2. Comparison the protective effects between artificial mucus and sputum samples. Scatter plots for tests performed using artificial mucus and sputum samples were merged (Figs. 3A and 5A) to compare protective capacities of the two types of mucus.

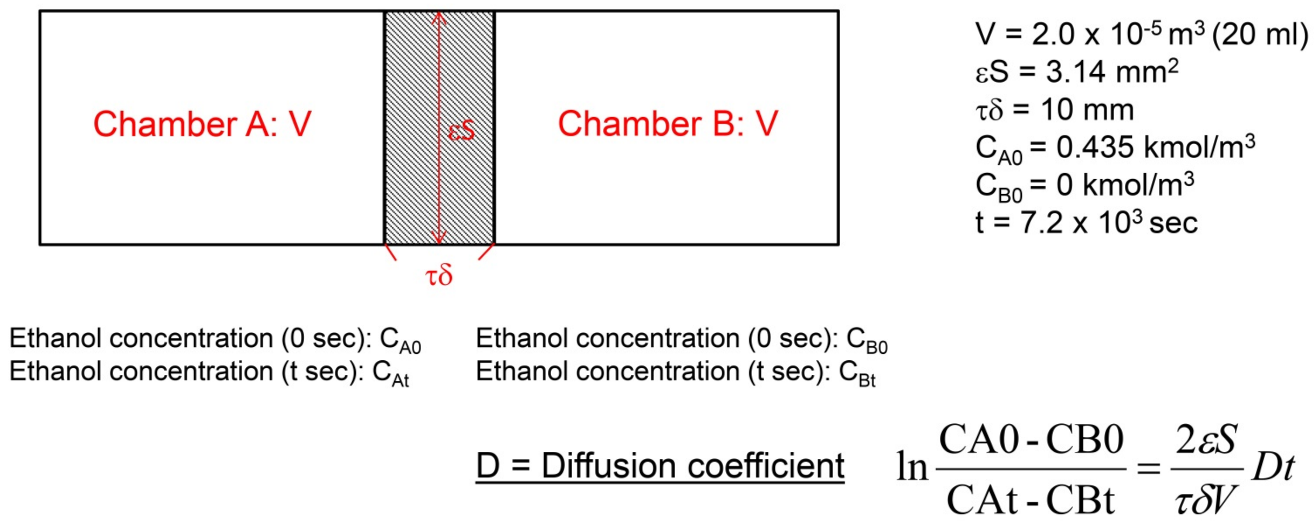

Supplementary Figure S3. Experimental setup to evaluate the diffusion rate of ethanol. A polyethylene container with two chambers (A and B) was used for the experiment. Changes in ethanol concentration in chambers A and B due to diffusion were measured to calculate the diffusion coefficient of ethanol. Chamber A was filled with ethanol, and chamber B was filled with xanthan gum-based artificial mucus, saline, or water. After 120 minutes of diffusion, the ethanol concentration in each chamber was measured, and the diffusion coefficient calculated.

Supplementary Table 1. Viscosity and titre ratio (IAV) of sputum samples.

	Viscosity (Pa·s)	Titre Ratio (IAV), mean ± SD		
		80% Ethanol	70% 2-Propanol	60% 1-Propanol
**saline**	1.0×10^{-3}	0.0 ± 0.0	0.0 ± 0.0	0.0 ± 0.0
**Sample 1**	5.2×10^{0}	$5.7 \times 10^{-1} \pm 7.2 \times 10^{-3}$	$5.6 \times 10^{-1} \pm 7.3 \times 10^{-2}$	$1.4 \times 10^{-1} \pm 6.9 \times 10^{-3}$
**Sample 2**	1.5×10^{1}	$6.9 \times 10^{-1} \pm 2.6 \times 10^{-2}$	$6.5 \times 10^{-1} \pm 3.7 \times 10^{-3}$	$3.0 \times 10^{-1} \pm 1.1 \times 10^{-2}$
**Sample 3**	1.1×10^{1}	$6.0 \times 10^{-1} \pm 2.0 \times 10^{-2}$	$5.8 \times 10^{-1} \pm 2.0 \times 10^{-2}$	$1.5 \times 10^{-1} \pm 4.3 \times 10^{-3}$
**Sample 4**	2.3×10^{1}	$7.3 \times 10^{-1} \pm 4.6 \times 10^{-2}$	$7.2 \times 10^{-1} \pm 1.4 \times 10^{-2}$	$3.2 \times 10^{-1} \pm 1.9 \times 10^{-2}$
**Sample 5**	2.0×10^{1}	$6.9 \times 10^{-1} \pm 1.7 \times 10^{-2}$	$6.5 \times 10^{-1} \pm 2.4 \times 10^{-2}$	$1.9 \times 10^{-1} \pm 8.1 \times 10^{-3}$
**Sample 6**	1.7×10^{1}	$7.3 \times 10^{-1} \pm 1.2 \times 10^{-2}$	$6.9 \times 10^{-1} \pm 3.0 \times 10^{-2}$	$3.2 \times 10^{-1} \pm 7.2 \times 10^{-3}$
**Sample 7**	1.7×10^{1}	$7.4 \times 10^{-1} \pm 2.3 \times 10^{-2}$	$7.2 \times 10^{-1} \pm 3.1 \times 10^{-2}$	$3.8 \times 10^{-1} \pm 2.1 \times 10^{-2}$
**Sample 8**	1.5×10^{1}	$7.9 \times 10^{-1} \pm 4.2 \times 10^{-2}$	$7.5 \times 10^{-1} \pm 3.7 \times 10^{-2}$	$3.2 \times 10^{-1} \pm 1.7 \times 10^{-2}$
**Sample 9**	2.1×10^{1}	$7.7 \times 10^{-1} \pm 4.4 \times 10^{-2}$	$7.0 \times 10^{-1} \pm 1.3 \times 10^{-2}$	$4.0 \times 10^{-1} \pm 2.7 \times 10^{-2}$
**Sample 10**	7.4×10^{0}	$4.8 \times 10^{-1} \pm 1.5 \times 10^{-2}$	$4.6 \times 10^{-1} \pm 1.9 \times 10^{-2}$	$1.7 \times 10^{-1} \pm 2.8 \times 10^{-2}$
**Sample 11**	1.8×10^{1}	$7.1 \times 10^{-1} \pm 1.5 \times 10^{-2}$	$6.6 \times 10^{-1} \pm 1.4 \times 10^{-2}$	$3.1 \times 10^{-1} \pm 2.7 \times 10^{-3}$
**Sample 12**	1.5×10^{1}	$5.8 \times 10^{-1} \pm 3.3 \times 10^{-2}$	$5.7 \times 10^{-1} \pm 2.1 \times 10^{-2}$	$2.5 \times 10^{-1} \pm 6.9 \times 10^{-3}$
**Sample 13**	1.4×10^{1}	$5.6 \times 10^{-1} \pm 1.6 \times 10^{-2}$	$5.5 \times 10^{-1} \pm 2.5 \times 10^{-2}$	$2.8 \times 10^{-1} \pm 2.0 \times 10^{-2}$
**Sample 14**	1.3×10^{1}	$5.9 \times 10^{-1} \pm 2.4 \times 10^{-2}$	$5.7 \times 10^{-1} \pm 5.3 \times 10^{-2}$	$2.1 \times 10^{-1} \pm 1.0 \times 10^{-2}$
**Sample 15**	1.2×10^{1}	$6.4 \times 10^{-1} \pm 4.3 \times 10^{-2}$	$6.1 \times 10^{-1} \pm 1.6 \times 10^{-2}$	$3.8 \times 10^{-1} \pm 4.4 \times 10^{-4}$
**Sample 16**	2.1×10^{1}	$7.4 \times 10^{-1} \pm 2.1 \times 10^{-2}$	$7.1 \times 10^{-1} \pm 4.0 \times 10^{-3}$	$4.0 \times 10^{-1} \pm 1.3 \times 10^{-2}$
**Sample 17**	2.6×10^{1}	$7.9 \times 10^{-1} \pm 1.9 \times 10^{-2}$	$7.3 \times 10^{-1} \pm 1.9 \times 10^{-2}$	$4.0 \times 10^{-1} \pm 2.0 \times 10^{-2}$
**Sample 18**	1.9×10^{1}	$7.4 \times 10^{-1} \pm 3.8 \times 10^{-2}$	$7.6 \times 10^{-1} \pm 3.6 \times 10^{-2}$	$3.6 \times 10^{-1} \pm 2.6 \times 10^{-2}$
**Sample 19**	1.5×10^{1}	$6.6 \times 10^{-1} \pm 5.7 \times 10^{-2}$	$6.0 \times 10^{-1} \pm 1.2 \times 10^{-2}$	$2.0 \times 10^{-1} \pm 4.0 \times 10^{-3}$
**Sample 20**	1.3×10^{1}	$6.7 \times 10^{-1} \pm 7.6 \times 10^{-3}$	$6.7 \times 10^{-1} \pm 7.4 \times 10^{-3}$	$3.0 \times 10^{-1} \pm 1.2 \times 10^{-2}$
**Sample 21**	1.4×10^{1}	$6.5 \times 10^{-1} \pm 5.5 \times 10^{-2}$	$6.2 \times 10^{-1} \pm 3.4 \times 10^{-2}$	$2.6 \times 10^{-1} \pm 6.5 \times 10^{-3}$
**Sample 22**	1.6×10^{1}	$6.5 \times 10^{-1} \pm 6.0 \times 10^{-2}$	$6.1 \times 10^{-1} \pm 5.0 \times 10^{-2}$	$2.8 \times 10^{-1} \pm 1.1 \times 10^{-2}$
**Sample 23**	2.3×10^{1}	$7.2 \times 10^{-1} \pm 4.0 \times 10^{-2}$	$7.2 \times 10^{-1} \pm 2.5 \times 10^{-2}$	$3.8 \times 10^{-1} \pm 3.0 \times 10^{-2}$
**Sample 24**	1.6×10^{0}	$4.0 \times 10^{-1} \pm 1.1 \times 10^{-2}$	$3.9 \times 10^{-1} \pm 2.0 \times 10^{-2}$	$1.1 \times 10^{-1} \pm 1.1 \times 10^{-2}$
**Sample 25**	1.7×10^{0}	$4.1 \times 10^{-1} \pm 1.0 \times 10^{-2}$	$3.9 \times 10^{-1} \pm 1.0 \times 10^{-2}$	$1.1 \times 10^{-1} \pm 1.4 \times 10^{-2}$
**Sample 26**	1.6×10^{0}	$4.0 \times 10^{-1} \pm 1.2 \times 10^{-2}$	$3.9 \times 10^{-1} \pm 1.4 \times 10^{-2}$	$1.1 \times 10^{-1} \pm 1.4 \times 10^{-2}$
**Sample 27**	4.8×10^{0}	$5.4 \times 10^{-1} \pm 1.2 \times 10^{-2}$	$5.2 \times 10^{-1} \pm 1.5 \times 10^{-2}$	$1.5 \times 10^{-1} \pm 9.1 \times 10^{-3}$

Supplementary Table 2. Viscosity and titre ratio (IAV) of pronase-treated sputum samples.

	Viscosity (Pa·s)	Titre Ratio (IAV), mean ± SD		
		80% Ethanol	70% 2-Propanol	60% 1-Propanol
**saline**	1.0×10^{-3}	0.0 ± 0.0	0.0 ± 0.0	0.0 ± 0.0
**Sample 1**	7.6×10^{-2}	$3.4 \times 10^{-2} \pm 5.5 \times 10^{-4}$	$3.3 \times 10^{-2} \pm 1.3 \times 10^{-3}$	0.0 ± 0.0
**Sample 2**	1.6×10^{-2}	0.0 ± 0.0	0.0 ± 0.0	0.0 ± 0.0
**Sample 3**	5.0×10^{-2}	0.0 ± 0.0	0.0 ± 0.0	0.0 ± 0.0
**Sample 4**	4.2×10^{-2}	0.0 ± 0.0	0.0 ± 0.0	0.0 ± 0.0
**Sample 5**	2.1×10^{-2}	0.0 ± 0.0	0.0 ± 0.0	0.0 ± 0.0
**Sample 6**	3.4×10^{-2}	0.0 ± 0.0	0.0 ± 0.0	0.0 ± 0.0
**Sample 7**	2.3×10^{-2}	0.0 ± 0.0	0.0 ± 0.0	0.0 ± 0.0
**Sample 8**	4.1×10^{-2}	0.0 ± 0.0	0.0 ± 0.0	0.0 ± 0.0
**Sample 9**	4.9×10^{-2}	0.0 ± 0.0	0.0 ± 0.0	0.0 ± 0.0
**Sample 10**	2.4×10^{-2}	0.0 ± 0.0	0.0 ± 0.0	0.0 ± 0.0
**Sample 11**	3.3×10^{-2}	0.0 ± 0.0	0.0 ± 0.0	0.0 ± 0.0
**Sample 12**	4.9×10^{-2}	0.0 ± 0.0	0.0 ± 0.0	0.0 ± 0.0
**Sample 13**	3.6×10^{-2}	0.0 ± 0.0	0.0 ± 0.0	0.0 ± 0.0
**Sample 14**	1.6×10^{-2}	0.0 ± 0.0	0.0 ± 0.0	0.0 ± 0.0
**Sample 15**	3.0×10^{-2}	0.0 ± 0.0	0.0 ± 0.0	0.0 ± 0.0
**Sample 16**	4.7×10^{-2}	0.0 ± 0.0	0.0 ± 0.0	0.0 ± 0.0
**Sample 17**	3.6×10^{-2}	0.0 ± 0.0	0.0 ± 0.0	0.0 ± 0.0
**Sample 18**	4.9×10^{-2}	0.0 ± 0.0	0.0 ± 0.0	0.0 ± 0.0
**Sample 19**	4.6×10^{-2}	0.0 ± 0.0	0.0 ± 0.0	0.0 ± 0.0
**Sample 20**	6.3×10^{-2}	0.0 ± 0.0	0.0 ± 0.0	0.0 ± 0.0
**Sample 21**	3.3×10^{-2}	0.0 ± 0.0	0.0 ± 0.0	0.0 ± 0.0
**Sample 22**	7.0×10^{-2}	0.0 ± 0.0	0.0 ± 0.0	0.0 ± 0.0
**Sample 23**	7.2×10^{-2}	$2.9 \times 10^{-2} \pm 1.0 \times 10^{-3}$	$2.8 \times 10^{-2} \pm 1.5 \times 10^{-3}$	0.0 ± 0.0
**Sample 24**	1.7×10^{-2}	0.0 ± 0.0	0.0 ± 0.0	0.0 ± 0.0
**Sample 25**	3.3×10^{-2}	0.0 ± 0.0	0.0 ± 0.0	0.0 ± 0.0
**Sample 26**	5.1×10^{-2}	0.0 ± 0.0	0.0 ± 0.0	0.0 ± 0.0
**Sample 27**	7.0×10^{-2}	0.0 ± 0.0	0.0 ± 0.0	0.0 ± 0.0

Supplementary Table 3. Titre ratios were compared between pronase-treated and untreated groups based on inactivation test results.

	Saline	Sputum	Sputum + Pronase	*P* value*
Sample number		27	27	
Viscosity, Pa·s, mean ± SD	0.001	14.1 ± 6.7	0.040 ± 0.018	<0.001
Titre ratio, mean ± SD (80% Ethanol)	0.0 ± 0.0	0.64 ± 0.12	0.002 ± 0.008	<0.001
Titre ratio, mean ± SD (70% 2-Propanol)	0.0 ± 0.0	0.61 ± 0.11	0.002 ± 0.008	<0.001
Titre ratio, mean ± SD (60% 1-Propanol)	0.0 ± 0.0	0.27 ± 0.10	0.0 ± 0.0	<0.001

* *P* value; Sputum vs. Sputum + Pronase

Supplementary Table 4. Resistance to UV radiation of IAV in sputum samples with or without pronase treatment.

	Saline	Sputum	Sputum + Pronase	*P* value*
Sample number		27	27	
Viscosity, Pa·s, mean ± SD	0.001	14.1 ± 6.7	0.040 ± 0.018	<0.001
Titre ratio, mean ± SD (UV, 0 mJ/cm^2)	1.0 ± 0.0	1.0 ± 0.0	1.0 ± 0.0	
Titre ratio, mean ± SD (UV, 2.25 mJ/cm^2)	$1.5 \times 10^{-2} \pm 2.0 \times 10^{-4}$	$1.4 \times 10^{-2} \pm 2.3 \times 10^{-4}$	$1.4 \times 10^{-2} \pm 2.9 \times 10^{-4}$	0.059
Titre ratio, mean ± SD (UV, 4.5 mJ/cm^2)	$1.0 \times 10^{-3} \pm 9.6 \times 10^{-5}$	$9.7 \times 10^{-4} \pm 2.9 \times 10^{-5}$	$9.7 \times 10^{-4} \pm 2.1 \times 10^{-5}$	0.776
Titre ratio, mean ± SD (UV, 6.75 mJ/cm^2)	0.0 ± 0.0	0.0 ± 0.0	0.0 ± 0.0	

* *P* value; Sputum vs. Sputum + Pronase
